# Determinants of Malnourishment in the Institutionalized Older Population: The FRAGILESS Study

**DOI:** 10.3390/nu16234114

**Published:** 2024-11-28

**Authors:** Julia Leira, Ana Maseda, Rocío López-López, Laura Lorenzo-López, Nuria Cibeira, Leire Lodeiro-Fernández, José C. Millán-Calenti

**Affiliations:** Universidade da Coruña, Gerontology and Geriatrics Research Group, Instituto de Investigación Biomédica de A Coruña (INIBIC), Complexo Hospitalario Universitario de A Coruña (CHUAC), SERGAS, 15071 A Coruña, Spain; julia.leira.morano@udc.es (J.L.); rocio.lopez.lopez@udc.es (R.L.-L.); laura.lorenzo.lopez@udc.es (L.L.-L.); nuria.cibeira@udc.es (N.C.); leire.lodeiro@udc.es (L.L.-F.); jcmillan@udc.es (J.C.M.-C.)

**Keywords:** malnutrition, undernutrition, Mini Nutritional Assessment, elderly

## Abstract

Background/Objectives: Malnutrition is a very common condition among older people and strongly affects their quality of life. The current literature relates the presence of nutritional deficiencies to several health-related factors that usually emerge at advanced stages of life. This study aimed to assess the associations between malnutrition and its determinants in a group of institutionalized older people via the Mini Nutritional Assessment–Short Form (MNA-SF) and the full MNA. Methods: The MNA-SF was compared with the full MNA to evaluate the nutritional status of 207 older people. A multinomial logistic regression analysis was performed. Results: The data revealed that institutionalized older people with cognitive impairment, frailty syndrome, dysphagia, a low BMI, a high duration of institutionalization, and a low educational level are more likely to be malnourished or at risk of malnutrition. Conclusions: The results reveal that the MNA or MNA-SF may not identify common determinants of malnutrition or nutritional risk. The identified determinants depend on the test. Therefore, the data obtained determine the need to use adequate nutritional screening tools to control the presence of malnutrition. Nutritional screening is essential to decrease public costs, hospitalizations, rates of disability, dependence, morbidity, and even mortality among institutionalized older people.

## 1. Introduction

According to the World Health Organization (WHO), malnutrition refers to “deficiencies or excesses in nutrient intake, imbalance of essential nutrients or impaired nutrient utilization” [1]. Malnutrition is a global public health problem and very prevalent in developed countries. Current evidence affirms that the risk of malnutrition is much greater among older individuals [2,3]. The WHO clarifies that malnutrition includes malnutrition, vitamin or mineral imbalance, overweight, obesity, and diseases that compromise nutrition. The percentage of older adults with problems of overweight and obesity is approximately 45%, whereas 462 million older adults experience moderate or severe malnutrition [1]. Malnutrition percentages are approximately 12–50%, specifically in hospitalized people, and 23–60% in institutionalized older people [4].

As mentioned, malnutrition is very common among older adults. The presence of this condition may be partly due to the physiological decline that humans suffer at this stage of life, the appearance of certain degenerative diseases, or the decrease in intake of food and essential nutrients [2]. Malnutrition deteriorates the quality of life and significantly increases public spending for its treatment, progressively increases hospitalization and increases the mortality rate [2,5,6,7,8]. The results of a systematic review of six longitudinal studies demonstrated that age was a risk factor for the development of malnutrition [9]. Specifically, malnutrition is also very common in older people with a good state of health [10].

The percentage of malnutrition considerably increases in older people in situations of isolation, dependence, and depression [4]. In cases of malnutrition, older people may experience an increased risk of falls, a substantial reduction in autonomy, gait disorders, and problems with healing wounds [11]. In addition, other very common pathologies or conditions at advanced ages represent risk factors for malnutrition, such as frailty in institutionalized people, polypharmacy, cognitive impairment, deterioration in general health, Parkinson’s disease, institutionalization, and the presence of dysphagia or swallowing abnormalities [9,12]. A study revealed that dementia and Alzheimer’s disease (AD) were less common in older people with a correct nutritional status [13]. In this sense, malnutrition can be linked to emotional factors such as the presence of depression [14,15]. Low food and nutrient intake may be more specifically due to the feeling of loss of an active social role, deprivation of affection, or depression, which may be caused by institutionalization. In other words, emotional problems largely stem from feelings of uselessness, a sedentary lifestyle or the passive role of older people [15,16].

Meanwhile, malnutrition is also linked to functional and physical factors such as sarcopenia and frailty [17]. Cognitive impairment has been shown to correlate to an incorrect nutritional status among older people [12,18]. Socio-environmental factors must be considered. The percentage of malnutrition often increases if there are physical barriers, great distances between homes and places where food is supplied, etc. An older person may not be able to access fresh products for proper nourishment for many reasons [19].

The current literature emphasizes the importance of early attention to malnutrition in older people. The aforementioned risk factors should be considered for early detection. Additionally, they can offer strategies for individualized and specific identification and treatment. In addition, they can lead to the creation of prevention techniques and instruments [2,9].

Currently, the methods used to evaluate the nutritional status of older adults are complicated and time-consuming [20]. Specifically, the Mini Nutritional Assessment (MNA) and its short version (MNA-SF) are excellent instruments to assess the nutritional status of older people. It is a very complete, standardized, and validated instrument that also predicts the risk of hospitalization and even mortality [20,21]. However, the use of malnutrition detection instruments, the correct application of diagnostic criteria, quantification of protein deficiencies and potential causes of malnutrition, and the assessment of individual resources are necessary throughout this process [22]. Nevertheless, the MNA contains questions to self-evaluate nutritional and health status, which may reduce its applicability in patients with dementia or impaired speech capacity [23,24]. Questions that address food choices, portion size, and mode of feeding may also not be appropriate for patients who are nutritionally stable. Thus, MNA cannot be used in patients who receive enteral nutrition [24]. MNA and MNA-SF are useful tools to detect the likelihood of undernutrition in frail older adults and malnutrition in the early stages with good reliability and sensitivity [25].

Early treatment can improve the quality of life for older people [3,11]. Eating a balanced diet, consuming the right amount of foods rich in proteins, vitamins, and minerals, and adequate intake of water constitute a good basis to treat and prevent eating problems [11]. Therefore, the hypothesis is that nutritional status can be linked to the development of health-related parameters in older adults. A high percentage of older people report this type of problem, so the need to promote new lines of research that address these types of risk factors, which are linked to the evolution and development of nutritional problems, is becoming increasingly evident. The identification of all of these factors will be beneficial for the creation of action protocols to improve the quality of life of this group and reduce the health costs, dependence, disability, and mortality rates [26].

Thus, the main objective of this study was to examine the relationship of a series of multi-dimensional risk factors related to the health and nutritional status in institutionalized older people and to compare the prevalence of malnutrition and the identification of its determinants using MNA-SF with that using the full MNA.

## 2. Materials and Methods

### 2.1. Design

A comparative, descriptive, and cross-sectional study was performed on a sample of older people aged ≥ 65 years, who were recruited from a gerontological complex in Galicia (Spain). All participants, or their relatives in the case of inability, gave their written informed consent. The research protocol was reviewed and approved by the Autonomous Research Ethics Committee of Galicia (Spain). The study was conducted following the ethical standards in the Declaration of Helsinki.

### 2.2. Participants

The study participants were recruited from a gerontological center, where different qualified professionals are in charge of the care for older people, most of whom have frailty or dependence problems. The center has several places for daytime stays and other places for permanent stays.

In total, 207 older adults participated in the study. The participants were selected based on the following inclusion criteria: (a) aged 65 years or older and (b) agreed to and signed the informed consent form. Participants who were unable to complete the assessment measures were excluded.

### 2.3. Instruments and Outcome Measures

All participants underwent a Comprehensive Gerontological Assessment (VGI). The objective was to globally assess the nutritional, physical functioning, psychological, and social status of each individual. The assessments were performed by a multi-disciplinary team composed of nutritionists, occupational and speech therapists, psychologists, and nurses. Information on the gender, age, educational level, length of institutionalization (months), or use of chewing aids was reported.

To perform the nutritional assessment, the Mini Nutritional Assessment (MNA) [27] and Mini Nutritional Assessment Short Form (MNA-SF) [28] scales were used. These tools reveal the current nutritional status of each participant. In addition, they classify each individual in a state of malnutrition, risk of malnutrition, or well-nourished according to the score obtained from the test, and the cutoff points are different in the long version and short version (For MNA, scores < 17 of 30 indicate “undernutrition”, scores 17–23.5 indicate “at risk of malnutrition” and scores > 23.5 indicate “normal/well nourished”. For MNA-SF, scores of 0–7 indicate “malnourishment”, scores 8–11 indicate “at risk of malnutrition” and scores 12–14 indicate “normal”). It is especially designed for older people and widely used in care settings. Currently, it is available in several languages and is a good predictor of mortality and hospital costs due to malnutrition [29].

Weight and height measurements were obtained by a trained nurse. The BMI was estimated by dividing the weight (kilograms) by the square of the height (in meters). A clothing adjustment of approximately 0.8 kg for women and 1.2 kg for men was made [30]. The degree of comorbid illness was measured at the baseline using the Charlson Comorbidity Index (CCI) [31]. The age-adjusted CCI was computed.

The Spanish versions of the Mini-Mental State Examination (MMSE) [32] and severe MMSE (sMMSE) [33] were used as screening tools for dementia and cognitive impairment. MMSE scores of 0–30 were adjusted for the age and level of education considering cognitive impairment with scores ≤ 24. This tool enables rapid detection of mild or moderate cognitive impairment in clinical, community, and research contexts. Currently, it is the best-known and most commonly used method [34]. The sMMSE score range is also 0–30, where lower scores indicate greater impairment, and sMMSE is used when patients score 10 or less on the MMSE.

In addition, the Geriatric Depression Scale-Short Form (GDS-15) [35] was used to check for depressive symptomatology among the study participants, and a cutoff of 5 or higher was used to consider the existence of probable clinical depression. This scale, which was designed specifically for the geriatric population, explores the presence of depressive symptoms through 15 easy-to-understand questions with a dichotomous response pattern to facilitate its completion by the person being evaluated. The Cornell Scale for Depression in Dementia (CSDD) [36,37] was used to evaluate symptoms of major depression in patients with dementia by interviewing the patients and their formal caregivers. A cutoff point below 6 was considered the absence of significant depressive symptoms. Analysis of prescribed medications was based on the medical register. The Anatomical Therapeutic Chemical (ATC) classification was used to categorize the medications [38].

The study participants were classified as frail and pre-frail (non-frail or robust participants were not identified in our sample) according to Fried’s frailty criteria [39]. These criteria assess the unintentional weight loss, self-reported exhaustion, slow walking speed, weakness (grip strength), and low physical activity of each individual. If 3 or more different domains are met, individuals are classified as frail; if 1 or 2 frailty domains are met, they are classified as pre-frail.

The Swallowing Performance Scale (SPS) [40] was used to detect dysphagia. The scale quantifies dysphagia with a score of 1–7 according to the criteria of oral impairment, pharyngeal impairment, aspiration, and diet. A higher score indicates more severe dysphagia [41]. In this study, a score of 3 or higher was considered dysphagia.

### 2.4. Statistical Analysis

The baseline characteristics of the sample were analyzed using descriptive statistics to compare the distributions of participant characteristics for each variable. Chi-square tests and one-way analysis of variance (ANOVA) were used to detect possible differences according to the nutritional status (normal/well-nourished, at risk of malnutrition or malnutrition) after the administration of the Mini Nutritional Assessment (MNA) or MNA-SF. Column proportions were used for the pairwise comparisons of categorical variables. In addition, Spearman’s correlation coefficients were calculated between the independent variables and the total scores of the MNA and MNA-SF.

To determine the associations between the characteristics of the participants and their nutritional status (normal/well-nourished, at risk of malnutrition or malnutrition), which was determined by the administration of the MNA or MNA-SF, a multinomial logistic regression analysis was performed. Additionally, odds ratios (ORs) were estimated, and 95% confidence intervals (CIs) were calculated after adjustment for covariates. All statistical analyses were performed with IBM SPSS Statistics v.27.0 and R statistical software v.3.6.1 (using the R packages Rcmdr, MASS, and nparLD).

## 3. Results

### 3.1. Total Sample Characteristics

In total, 207 people were included in this study, 149 of whom were women, i.e., 72% of the sample. The mean age was 84.5 ± 7.8 years. The percentage of people who had received 8 or fewer years of education was 46.3%, 41.1% had 9–17 years of education, and only 12.6% had 18 or more years of education. The average length of institutionalization in the gerontological complex until the moment of data collection was 20.9 ± 24.1 months.

The MNA or MNA-SF was used to assess the nutritional status. After applying the MNA, a mean score of 20.3 ± 4.3 was obtained, and after applying the MNA-SF, a mean score of 9.7 ± 2.6 was obtained, which indicates that the majority of the sample was at risk of malnutrition according to both tests (no significant differences were found between the nutritional categories classified by MNA and MNA-SF, *p* = 0.376). Normal-status or well-nourished patients were present in only 25% of the sample, 60% were at risk of malnutrition, and approximately 15–20% presented malnourishment.

The mean BMI was 26.2 ± 5.7, and the age-adjusted CCI score was 6.2 ± 1.6. In total, 160 participants (78.8% of the sample) presented cognitive impairment, and, depression was observed in 44.2% of all participants. The mean number of drugs ingested per day was 8.4 ± 3.7. Frailty and pre-frailty criteria were observed in 68.6% and 31.4% of the sample, respectively. Furthermore, 65.7% used a type of chewing aid, and 33.2% suffered from swallowing problems.

### 3.2. Differences Between Multi-Dimensional Factors (Nutritional Status and MNA Score vs. MNA-SF Score)

The extended version of the MNA (Table 1) showed significant differences (*p* = 0.008) in the nutritional status by gender. The column proportions showed significant differences between normal individuals and those at risk of malnutrition, and women were at higher risk than men (79.3% vs. 20.7%). However, when the MNA-SF was used (Table 2), no significant differences (*p* = 0.312) were observed. The worst data in terms of nutrition were obtained from the examined women, but they were not significantly relevant. No significant differences were observed between the different nutritional statuses and the MNA versions in terms of age, level of education, age-adjusted CCI score, or use of chewing aids.

The length of institutionalization and number of drugs consumed significantly differed among the categories established by the MNA-SF. Patients who lived for more months in the nursing home were at risk of malnutrition (*p* < 0.001), and the number of drugs consumed decreased when the nutritional status worsened (9.0, 8.3, and 7.4, respectively, *p* = 0.039).

With respect to cognitive deficits, the tendency was similar between the MNA versions. Cognitive deficits were observed in 52–58.2% of cases with normal nutrition, 82.5–84.7% of cases at risk of malnutrition and 97.1–100% in malnutrition cases (*p* < 0.001). These percentages are not significantly different between those at risk of malnutrition and the malnourished.

Depressive symptoms occurred in 50.9% of cases at risk of malnutrition assessed by MNA, whereas they occurred in 26.5% of individuals with a normal nutritional status (*p* = 0.015). No significant differences were found in the presence of undernutrition assessment by the MNA-SF (*p* = 0.161).

Regarding the frailty phenotype, the trend was similar between both MNA versions: 81.8–91.2% of participants with malnutrition presented some criteria of frailty, as did 73.3–75.2% of those who presented an established risk of malnutrition and 42.0–46.3% of those who maintained a normal nutritional status (*p* < 0.001). The differences were not significant between risk of malnutrition and malnourishment.

Finally, the presence of dysphagia was similar between the two test versions, and there were significant differences between the groups. Dysphagia was detected in 60–69.4% of people with malnutrition, 30.6–32.8% of people at nutritional risk, and 12.5–16.7% of those with a normal nutritional status (*p* < 0.001).

Table 3 shows Spearman’s correlations between the studied variables and the MNA and MNA-SF scores. The correlations between cognitive impairment, frailty phenotype, and swallowing performance were negative and ranged from −0.258 to −0.439. In other words, worse cognitive and frailty status and the presence of swallowing problems were associated with lower MNA and MNA-SF scores. A positive correlation was found with BMI: better nutritional status appeared at higher BMIs. In addition, a lower number of drugs consumed (r = 0.154) and the use of chewing aids (r = −0.193) were associated with better MNA-SF scores.

The logistic regression analysis identified important determinants of malnourishment or the risk of malnutrition. Older people with cognitive impairment, frailty, and dysphagia were more than 30 times more likely to be malnourished or at risk of malnourishment (Table 4 and Table 5, with only significant variables included), although frailty was only identified using the full MNA analysis. In addition, as expected, low BMI is also a determinant of the risk of malnutrition or undernutrition. Finally, a longer duration of institutionalization and a lower educational level are determinants of malnutrition based on MNA-SF.

## 4. Discussion

To perform this study, the Mini Nutritional Assessment (MNA) and Mini Nutritional Assessment Short Form (MNA-SF) were used. The MNA is an excellent instrument for assessing the nutritional status of older adults [20,21], and the MNA-SF has greater sensitivity but lower specificity for patients aged over 65 years [42]. It is a validated and standardized screening tool that also helps to predict the hospitalization and mortality rates. However, Guyonnet et al. [3] reported that there were no standardized instruments to accurately detect malnutrition in this group; therefore, the malnutrition rate currently cannot be correctly estimated. Nevertheless, the MNA is a highly useful screening tool for detecting both nutritional risk and malnutrition, particularly in frail older people [43]. It is also ideal for routine use in health care and hospital settings [29]. The Short Nutritional Assessment Questionnaire (SNAQ), which is a screening tool used to predict the risk of malnutrition, can identify individuals who will lose weight earlier than MNA, but this scale is still rarely used. Currently, these scales are not used to detect nutritional problems in older adults because they require much time. The development of short versions aims to overcome this time limitation in professionals [3]. The Malnutrition Universal Screening Tool (MUST) was also identified as the most accurate nutritional screening tool for hospitalized patients in many clinical settings [42].

The percentage of malnutrition is the object of interest in this study. Hazzard et al. [4] reported a greater risk of malnutrition among older people who resided in a community. The reason may be situations of social isolation, self-imposed and environmental barriers and limitations. The barriers experienced by older people living in the community limit their access to food, which directly results in nutritional decline. Furthermore, the authors concluded that institutionalized people were subject to continuous medical care, unlike adults who live in the community. However, the rates reported by Kieswetter [6] of malnutrition among users of nursing homes and gerontological centers were very high (23–60%). Faravo-Moreira et al. [9] also noted that a person living in an institutional setting and in a situation of frailty was at high risk of malnutrition. Moreira et al. [44] affirmed that malnutrition was independently associated with residing in an institutionalized environment. Moreover, malnutrition is independent from the environment where a person is located and can occur in any scenario, except for those who live in the community [45]. In contrast, Donini et al. [16] explained that malnutrition in a community was frequent and continued to frequently occur in the most delicate and under-resourced groups of the population, e.g., older adults. Thus, they also stated that the malnutrition rate was higher among users of residences and institutional complexes. The reported data are as follows: 42.5% of women living in nursing homes suffer from malnutrition, whereas the percentage of older women with malnutrition living in a community is 14.5%. In the case of men, 30.8% of those living in nursing homes suffer from malnutrition, and only 2% of men living in a community are malnourished, which represents a great contrast between areas.

A study performed in residences for older people in Spain showed that malnutrition increased with age until 90 years of age, where the trend tends to stagnate. This rate has also been reported to be higher among older people institutionalized in residences in peripheral towns than among those in residences in the main cities [46]. Isenring et al. [47] reported that the nutritional risk of older adults living in a community was very low. Bell et al. [48] reported that approximately 20% of people who resided in nursing homes suffered from malnutrition. However, depending on the study, these rates are usually 1.5–66.5% of the total number of residents, so there is a wide range of variation between centers. Another study on public and private nursing homes in the city of Ankara [49] reported that 28.6% of residents suffered from malnutrition, and 44.5% were at risk of malnutrition. These alarming figures highlight a greater nutritional risk for nursing home users. However, another study with institutionalized older people in nursing homes in a rural area of Portugal [44] showed much lower percentages of malnutrition than expected in this context.

We observed a significant correlation between nutritional status and gender of the participants in the evaluation performed with the full MNA, but this correlation was not found in the regression model. The MNA-SF did not show this result because a study revealed that women were notably more likely to suffer from malnutrition than men, and this common difference is not understood today [50]. Therefore, although significant differences were found between several variables (such as gender or the presence of depressive symptoms) and nutritional status measured by MNA and/or MNA-SF, no evidence was found as determinants of malnutrition in the regression analysis.

A systematic review by O’Keeffe et al. [51] established conflicting evidence that depression was a determinant of malnutrition among older people, and it remains unclear whether depressive symptomatology is a cause or a consequence of malnutrition [52]. Yoshimura et al. [53] reported that depression and malnutrition tended to be closely related among young people but not among older people. Similarly, another study in China [54] reported that older people with malnutrition were 31% more likely to suffer from depression than older people who had a correct nutritional status. Our data revealed a significant difference only in the subjects evaluated by the full MNA and not in those assessed by MNA-SF.

In our study, no significant differences were detected between nutritional status and age or the use of chewing aids. Nevertheless, increased age can increase the risk of malnutrition due to physiological changes such as impaired taste, decreased gastric flexibility, and reduced appetite, which can exacerbate nutritional issues [52,55]. In addition, Moyihan et al. [56] reported that the use of specific aids such as dental prostheses could positively impact the nutritional status and cause greater enjoyment when eating. Hence, greater differences were observed in the data of people who use these aids and those who do not.

Low educational level and length of institutionalization were identified as determinants of malnourishment or risk of malnutrition only according to the MNA-SF. A meta-analysis revealed a significant relationship between low educational level and malnutrition or malnutrition risk, since individuals with higher education levels tend to have healthier and more diverse diets, which result in better nutritional status [57].

Our results identified important health-related parameters as determinants for malnutrition: frailty, cognitive impairment, and swallowing disorders. However, Bartali et al. [58] noted that low food intake was not related to the development of frailty among older people, and an association between malnutrition and/or the risk of malnutrition and frailty was found in a systematic review [59]. Favaro-Moreira et al. [9] established a decline in physical function as a very important risk factor for the development of malnutrition. Kieswetter [6] explained that if malnutrition was not correctly treated, the subject may be exposed to consequences at a physical functioning level. Another study reported that malnutrition detected by the MNA was significantly associated with functional measures based on the performance questionnaire [22]. Isenring et al. [47] reported that a higher rate of falls due to functional impairment was independently associated with nutritional risk. A study conducted in nursing homes in a Portuguese city [60] revealed that having cognitive decline and recurrent falls with associated injuries were not associated with poor nutritional status or risk of suffering from it. Our study revealed that cognitive impairment was also significantly associated with the poor nutritional status based on the full and short versions of the MNA. Kimura et al. [61] supported these data in their study and affirmed that the presence of malnutrition was very common among older adults with deterioration. In severe cognitive impairment, malnutrition may develop due to behavioral psychiatric symptoms of dementia (BPSD). A recent systematic review [12] mentioned possible causes of poor nutritional status and cognitive decline, since poor or deficient micronutrient intake plays key roles in the development and maintenance of brain structure and functions such as neurotransmitter activity, degeneration of nervous tissues and changes in brain functions. Another recent meta-analysis [62] revealed that older adults with cognitive frailty had a 3.77 times higher risk of malnutrition than those without cognitive frailty.

Swallowing disorders were also significantly associated with malnutrition status, as previously reported [63]. The consequences of the dysphagia–malnutrition relationship include weight loss, dehydration, muscle breakdown, fatigue, aspiration pneumonia, and a general decline in functional status [64]. Finally, our study did not suggest an association between the number of drugs consumed and malnutrition. Nevertheless, excessive polypharmacy was identified as a risk factor for malnutrition [9].

In summary, future studies should include the MNA and MNA-SF as ideal screening tools as part of a comprehensive assessment to identify multi-dimensional health factors that affect malnutrition. The nutritional status is an essential component to consider in long-term residential settings to establish an effective nutritional intervention, avoid increased morbidity and mortality and improve the quality of life of older people.

The main strength of this research is the large sample size, where data from institutionalized older people were comprehensively assessed. In addition, the analysis included potential health and social determinants. However, there are limitations, such as the cross-sectional nature of the study and the inability to determine the direction of the associations between the determinants and malnutrition or the risk of malnutrition. Furthermore, the limitations imposed by the descriptive nature of the study may hinder the generalization of the results. The Global Leadership Initiative on Malnutrition (GLIM) criteria were not published during the data collection; thus, they were not considered for diagnosing malnutrition [65].

## 5. Conclusions

In conclusion, this study analyzed the nutritional status and other health-related factors in a group of older people who resided in a gerontological center in a city in Spain. The results reveal that the MNA or MNA-SF can assess the nutritional status but do not identify the common determinants of malnutrition or nutritional risk. The relevant associated factors are cognitive impairment, frailty syndrome, dysphagia, low BMI, high length of institutionalization, and low educational level, depending on the test. Consequently, the findings highlight the need to use adequate nutritional screening tools to control the presence of malnutrition. This approach is crucial to reduce public costs, hospitalizations, rates of disability, dependence, morbidity, and mortality among institutionalized older people.

## Figures and Tables

**Table 1:** One-way ANOVA and chi-square tests were used to compare older people according to their nutritional status (Mini Nutrition Assessment (MNA)), normal/well-nourished (24–30 points), at risk of malnutrition (17–23.5 points), and undernutrition (less than 17 points).

	Normal/Well-Nourished (n = 50)		At risk of Malnutrition (n = 121)		Undernutrition (n = 36)			Total (n = 207)	
	N or Mean	% or SD	N or Mean	% or SD	N or Mean	% or SD	*p* Value	N or Mean	% or SD
Gender (n, %) (1)							0.008 **		
Men	22	44.0	25	20.7	11	30.6		58	28.0
Women	28	56.0	96	79.3	25	69.4		149	72.0
Age (years; mean, SD)	83.8	8.1	84.7	7.6	84.6	7.9	0.785	84,5	7.8
Education (years; n, %)							0.444		
≤8	28	56.0	51	42.1	17	47.2		96	46.3
9–17	18	36.0	54	44.7	13	36.1		85	41.1
≥18	4	8.0	16	13.2	6	16.7		26	12.6
Months institutionalized (mean, SD)	20.5	19.4	22.6	28.0	15.9	12.8	0,347	20.9	24.1
Total MNA score (mean, SD)	25.5	1.4	20.3	1.9	13.3	2.4	<0.001 ***	20.3	4.3
Total MNA-SF (mean, SD)	12.2	1.3	9.8	1.7	6.0	2.3	<0.001 ***	9.7	2.6
BMI (kg/m2; mean, SD)	28.4	4.7	26.8	5.8	21.6	3.7	<0.001 ***	26.2	5.7
Age-adjusted CCI score (mean, SD)	5.7	1.4	6.3	1.6	6.3	1.8	0.070	6.2	1.6
Cognitive impairment (MMSE or sMMSE; n, %) (2)							<0.001 ***		
Yes	26	52.0	100	84.7	34	97.1		160	78.8
No	24	48.0	18	15.3	1	2.9		43	21.2
Presence of depressive symptoms by GDS-SF or Cornell (n, %) (3)							0.015 *		
Yes	13	26.5	59	50.9	16	47.1		88	44.2
No	36	73.5	57	49.1	18	52.9		111	55.8
Number of drugs (mean, SD)	8.2	4.4	8.7	3.6	7.8	2.9	0.412	8.4	3.7
Frailty phenotype (n, %) (4)							<0.001 ***		
Prefrailty	29	58.0	32	26.7	3	8.8		64	31.4
Frailty	21	42.0	88	73.3	31	91.2		140	68.6
Chewing aids (n, %)							0.363		
Yes	37	74.0	76	62.8	23	63.9		136	65.7
No	13	26.0	45	37.2	13	36.1		71	34.3
Dysphagia (Swallowing Performance Scale; n, %) (5)							<0.001 ***		
Yes	6	12.5	37	30.6	25	69.4		68	33.2
No	42	87.5	84	69.4	11	30.6		137	66.8

BMI: body mass index; CCI: Charlson Comorbidity Index; GDS-SF: Geriatric Depression Scale–Short Form; MMSE: Mini-Mental State Examination; sMMSE: Severe Mini-Mental State Examination. * Significant (*p* value) < 0.05; ** significant (*p* value) < 0.01; *** significant (*p* value) < 0.001.

**Table 2:** One-way ANOVA and chi-square tests were used to compare older people according to their nutritional status (Mini Nutrition Assessment–Short Form, MNA-SF), normal status (12–14 points), risk of malnutrition (8–11 points) and malnourishment status (less than 7 points).

	Normal (n = 55)		At risk of Malnutrition (n = 117)		Malnourishment (n = 35)		
	N or Mean	% or SD	N or Mean	% or SD	N or Mean	% or SD	*p* Value
Gender (n, %)							0.312
Men	19	34.5	28	23.9	11	31.4	
Women	36	65.5	89	76.1	24	68.6	
Age (years; mean, SD)	83.8	7.7	84.8	7.9	84.2	7.1	0.749
Education (years; n, %)							0.179
≤8	32	58.2	50	42.7	14	40.0	
9–17	17	30.9	54	46.2	14	40.0	
≥18	6	10.9	13	11.1	7	20.0	
Months institutionalized (mean, SD)	18.9	17.9	23.7	28.6	13.7	10.9	<0.001 ***
Total MNA score (mean, SD)	24.6	2.3	20.3	2.5	13.8	3.1	0.043 *
Total MNA-SF (mean, SD)	12.7	0.8	9.6	1.0	5.4	1.7	<0.001 ***
BMI (kg/m^2^; mean, SD)	28.9	4.7	26.7	6.1	21.7	5.2	0.315
Age-adjusted CCI score (mean, SD)	5.9	1.4	6.2	1.6	6.4	1.8	0.533
Cognitive impairment (MMSE or sMMSE; n, %) (1)							<0.001 ***
Yes	32	58.2	94	82.5	34	100.0	
No	23	41.8	20	17.5	0	0.0	
Presence of depressive symptoms by GDS-SF or Cornell (n, %)							0.161
Yes	19	34.5	52	46.0	17	54.8	
No	36	65.5	61	54.0	14	45.2	
Number of drugs (mean, SD)	9.0	4.4	8.3	3.5	7.4	3.3	0.039 *
Frailty phenotype (n, %) (2)							<0.001 ***
Prefrailty	29	53.7	29	24.8	6	18.2	
Frailty	25	46.3	88	75.2	27	81.8	
Chewing aids (n, %)							0.079
Yes	41	74.5	77	65.8	18	51.4	
No	14	25.5	40	34.2	17	48.6	
Dysphagia (Swallowing Performance Scale; n, %) (3)							<0.001 ***
Yes	9	16.7	38	32.8	21	60.0	
No	45	83.3	78	67.2	14	40.0	

BMI: body mass index; CCI: Charlson Comorbidity Index; GDS-SF: Geriatric Depression Scale–Short Form; MMSE: Mini-Mental State Examination; sMMSE: Severe Mini-Mental State Examination. * Significant (*p* value) < 0.05; *** significant (*p* value) < 0.001.

**Table 3:** Spearman’s correlation coefficients among independent variables and Mini Nutritional Assessment (MNA) and MNA–Short Form (MNA-SF) total scores.

	MNA Total Score (max. 30 Points)	MNA-SF Score (max. 14 Points)
Gender	−0.082	−0.095
Age (years)	0.007	0.007
Education (years)	−0.084	−0.123
Months institutionalized	0.048	0.046
Total MNA Score		0.831 ***
Total MNA-SF score	0.831 ***	
BMI (kg/m^2^)	0.447 ***	0.494 ***
Age-adjusted CCI score	−0.079	−0.042
Cognitive impairment	−0.375 ***	−0.306 ***
Presence of depressive symptoms	−0.127	−0.117
Number of drugs	0.039	0.154 *
Frailty phenotype	−0.354 ***	−0.272 ***
Dysphagia	−0.439 ***	−0.258 ***
Chewing aids	0.122	−0.193 **

BMI: body mass index; CCI: Charlson Comorbidity Index. * Significant (*p* value) < 0.05; ** significant (*p* value) < 0.01; *** significant (*p* value) < 0.001.

**Table 4:** Results of the multinomial logistic regression analysis for factors associated with normal/well-nourished status or risk of malnutrition relative to undernutrition (Mini Nutritional Assessment, MNA) (χ2 = 112.268, *p* < 0.001; −2 log likelihood 198.19, *p* < 0.001; Cox and Snell R2 0.487; Nagelkerke R2 0.579; 76.8% of cases precisely predicted). Only variables that indicate significant effects are included.

	Normal/Well-Nourished vs. Undernutrition		At Risk of Malnutrition vs. Undernutrition			
	OR	95% CI	*p* Value	OR	95% CI	*p* Value
BMI	1.607	1.1312–1.967	<0.001 ***	1.473	1.220–1.779	<0.001 ***
Presence of cognitive impairment	83.958	2.105–3348.642	0.018 *			
Presence of frailty	32.886	3.957–273.278	0.001 **	8.308	1.179–58.555	0.034 *
Presence of swallowing difficulties	33.995	4.995–231.376	<0.001 ***	11.166	2.436–51.171	0.002 **

BMI: Body mass index. * Significant (*p* value) < 0.05; ** significant (*p* value) < 0.01; *** significant (*p* value) < 0.001.

**Table 5:** Results of the multinomial logistic regression analysis for factors associated with normal or risk of malnutrition relative to malnourishment (Mini Nutritional Assessment, MNA-SF) (χ2 = 107.964, *p* < 0.001; −2 log likelihood 215.64, *p* < 0.001; Cox and Snell R2 0.472; Nagelkerke R2 0.554; 72.8% of cases precisely predicted). Only variables that indicate significant effects are included.

	Normal vs. Malnourishment		At risk of Malnutrition vs. Malnourishment			
	OR	95% CI	*p* Value	OR	95% CI	*p* Value
BMI	1.731	1.374–2.181	<0.001 ***	1.586	1.271–1.980	<0.001 ***
Months institutionalized				1.074	1.013–1.139	0.017 *
Presence of cognitive impairment	9.2410	3.93510–2.25911	<0.001 ***			
Presence of swallowing difficulties	31.645	4.654–215.159	<0.001 ***	10.156	1.926–53.539	0.006 **
Low education (≤8 years)	0.025	0.002–0.317	0.004 **	0.059	0.006–0.583	0.015 *

BMI: Body mass index. * Significant (*p* value) < 0.05; ** significant (*p* value) < 0.01; *** significant (*p* value) < 0.001.

## Data Availability

The original contributions presented in the study are included in the article, further inquiries can be directed to the corresponding author.
